# Enhanced awareness of faces with slight downwards gazes in the breaking continuous flash suppression paradigm

**DOI:** 10.1007/s10339-025-01303-7

**Published:** 2025-10-22

**Authors:** Mayuna Ishida, Masaki Mori

**Affiliations:** 1 Graduate School of Media and Governance, Keio University, Endo 5322, Fujisawa, Kanagawa 252-0882 Japan; 2 Center for Data Science, Waseda University, Nishi-Waseda 1-6-1, Shinjuku, Tokyo 169-8050 Japan

**Keywords:** Continuous flash suppression, Face perception, Gaze perception, Downwards gaze

## Abstract

Previous studies have suggested that faces with direct gazes are perceived more quickly than are those with averted gazes under the breaking continuous flash suppression (b-CFS) paradigm. Although averted gazes are typically examined in the horizontal orientation (leftwards and rightwards), their effects in the vertical orientation (upwards and downwards) remain underexplored. To investigate how gaze direction influences face awareness and perception in both horizontal and vertical orientations under the b-CFS paradigm, 68 participants observed faces with downwards, upwards, leftwards, rightwards, or direct gazes. These faces were presented to one eye, while a dynamic Mondrian pattern was shown to the other through a binocular separator. The participants first detected the face and then identified its gaze direction. The results indicated that the detection time for faces with downwards gazes was shorter than was the detection time for faces with the other four gaze types. However, the accuracy of gaze discrimination for downwards gazes was lower than was that for the other four gaze types. These findings suggest that downwards gazes are perceived more easily but are discriminated less easily than other gaze directions are during unconscious face processing.

## Introduction

Eye information is key in social scenarios, as it indicates people’s interest, attention, and intention (Emery 2000; Hessels 2020; Kleinke 1986; Tomasello et al 2007). Compared with the eyes of other primates closely related to humans, such as chimpanzees, gorillas, and orangutans, human eyes have a unique morphology characterized by a large sclera, high contrast between the sclera/iris and surrounding skin tone, and a greater width/height ratio for the eye outline (Kobayashi and Kohshima 1997, 2001). These previous studies have suggested that the morphological features of the human eye serve as social signals in communicative contexts, enabling others to readily infer interest and attention from eye position. Therefore, understanding how the human eye affects gaze perception is essential for elucidating the mechanisms underlying human social cognition.

Eye position has been classified as either a direct or an averted gaze in previous social cognition studies. A direct gaze, in which a person truly looks straight at another person’s eyes, evokes the perception of eye contact and is closely associated with self-referential thinking (Baltazar et al 2014; Hietanen 2018; Stoyanova et al 2010). Conty et al (2016) propose watching eyes effects as a two-stage process for perceiving direct gazes: direct gazes attract attention (Stage 1), resulting in self-referential processing (Stage 2). In contrast, averted gazes, in which a person does not truly look straight at another person’s eyes, are useful for determining the focus of a person’s attention (Baron-Cohen et al 1995). Localization, detection, and discrimination are faster for objects that are frequently gazed at by another person than they are for objects that are not gazed at (Dalmaso et al 2020; Frischen et al 2007; McKay et al 2021, reviews of the gaze cueing effect). Thus, the classification of eye positions as direct or averted gazes reflects their distinct roles in social interactions, with both types of gaze serving as essential nonverbal signals that enable humans to effectively navigate social contexts.

Various studies have revealed that direct gazes attract more attention than averted gazes do (Conty et al 2007; Senju and Johnson 2009; Yokoyama et al 2011). For example, a previous study has reported that direct gazes hold attention longer than averted gazes do in free-view settings (Palanica and Itier 2012). Direct gazes are detected faster than averted gazes are in scenarios in which multiple faces are simultaneously presented, which is known as the stare-in-the-crowd effect (e.g., Conty et al, 2006; von Grünau and Anston, 1995; Shirama, 2012; Palanica and Itier, 2011; however, see Cooper et al, 2013; Framorando et al, 2016). The stare-in-the-crowd effect occurs not only for true direct gaze but also for faces perceived to be looking directly (Doi and Ueda 2007; Doi et al 2009), a perceptual configuration in which the gaze and head directions appear aligned towards the observer, as in the Wollaston illusion (Gibson and Pick 1963; Otsuka et al 2016; Wollaston 1824). Furthermore, in the gaze cueing paradigm, targets are detected more slowly when distractor stimuli are faces exhibiting a true or perceived direct gaze than they are when targets exhibit an averted gaze (McKay et al 2021; Friesen and Kingstone 1998; Senju and Hasegawa 2005). These studies suggest that true or perceived direct gazes attract more attention than averted gazes under conscious viewing conditions.

*Are true and perceived direct gazes more easily switched from unconscious to conscious than averted gazes?* The breaking continuous flash suppression (b-CFS) paradigm is often used to investigate this question. The (b-)CFS paradigm is a binocular rivalry method in which dynamic Mondrian stimuli are presented to one eye to suppress the visibility of stimuli presented to the other eye (Pournaghdali and Schwartz 2020; Tsuchiya and Koch 2005; Yang et al 2007, 2014). In the b-CFS paradigm, response time is often several seconds, which is generally longer than those observed in typical response time tasks such as face classification and object localization (e.g., Lanfranco et al 2022; Stein et al 2011). While debate is ongoing regarding whether the (b-)CFS paradigm inhibits low- or high-level processing (Moors et al 2017, 2019; Sklar et al 2018), the b-CFS paradigm, which primarily involves measuring the response time between stimulus presentation and perception, allows researchers to assess the accessibility, detectability, and relative strength of stimulus processing from an unconscious state to a conscious state (Gayet et al 2014; Stein and Sterzer 2014; Stein 2019). Indeed, faces with true or perceived direct gazes are detected faster than are those with averted gazes under the b-CFS paradigm (Akechi et al 2014; Chen and Yeh 2012; Chen et al 2022; Jackson and Seymour 2022; Lanfranco et al 2022; Seymour et al 2016; Stein et al 2011).

Averted gazes may be perceived as direct gazes within a certain range without considering head direction (Gamer and Hecht 2007; Lord and Haith 1974; Mareschal et al 2013). The perceptual region of the direct gaze differs between approximately $\pm 2$ degrees and $\pm 8$ degrees depending on several psychological factors, stimuli, and measurements (Balsdon and Clifford 2018; Horstmann and Linke 2022; Jun et al 2013; Linke and Horstmann 2024). Furthermore, gaze deviations have been investigated considering overestimation of the gaze direction, revealing that the gaze direction is perceived as more deviated than it actually is, particularly in the horizontal orientation (leftwards and rightwards;, Linke and Horstmann 2024; Otsuka and Clifford 2018; Palmer and Clifford 2018).

In the present study, the conscious and unconscious processing of slight gaze deviations in the vertical direction (upwards and downwards) were investigated. Several studies have explored the perception of gaze direction in the vertical orientation (Alais et al 2018; Anstis et al 1969; Horstmann 2025; Imai et al 2006; Mori and Watanabe 2018, 2020). These studies revealed that gaze deviations greater than 12 degrees in the vertical orientation are perceived as exaggerated. However, there is no consensus on whether gaze deviations of less than 12 degrees in the vertical orientation are perceived as underestimations (Cline 1967) or overestimations (Alais et al 2018). Although it is unclear whether the perceptual region associated with the direct gaze is anisotropic in the vertical orientation, the mode of the probability distribution for the perceptual region is approximately 2 degrees downwards (Horstmann and Linke 2022; Palmer et al 2022). On the basis of these findings, slightly deviated gaze directions appear to be perceived differently in the downwards orientation than they are in other directions. However, previous studies have not examined gaze directions beyond the direct, leftwards, and rightwards directions under the b-CFS paradigm. Furthermore, the question of how an individual becomes aware of a downwards gaze direction has not been directly investigated. If a slight downwards gaze is perceived as a direct gaze, the gaze may invoke the watching eye effect.

Considering the gap in understanding regarding gaze perception under the b-CFS paradigm and the recognition of slight gaze deviations in the vertical direction, this study aimed to investigate the effect of gaze direction on face awareness by comparing upwards, downwards, rightwards, leftwards, and direct gazes. We hypothesized that the perception of a slight downwards gaze would occur as quickly as that of a direct gaze in unconscious processing, on the basis of the finding that the centre of the eye contact zone has a slightly downwards gaze (Palmer et al 2022) and the effect of self-referential thinking (Baltazar et al 2014; Conty et al 2016; Hietanen 2018). Additionally, consistent with previous studies (e.g., Lanfranco et al 2022; Stein et al 2011; Yokoyama et al 2013), we predicted that a direct gaze is more likely to be detected than a leftwards or rightwards gaze is.

## Methods

### Participants

Seventy-six undergraduate and graduate students participated in the experiment. Eight participants were excluded from the analysis because of difficulty in perceiving the binocular stereo vision stimuli ($n = 6$), an insufficient understanding of the instructions for the face detection task ($n = 1$), or missing responses on key measures ($n = 1$). Thus, the number of participants with valid responses was 68 (38 females, 30 males). The participants’ mean age was 20.67 years ($SD = 1.87$ years; range $=$ 18–25). Although precise ethnic information was not accurately collected, all of the participants were affiliated with a Japanese university and were predominantly Japanese (Asian). The Miles test (Miles 1930) was performed to determine the dominant eye. Forty-four participants had a dominant right eye, and twenty-four participants had a dominant left eye.

A post hoc power analysis for the analysis of variance (ANOVA) design in this study was conducted using the Superpower package (version 0.2.2, Lakens and Caldwell 2021). The power was calculated with $\alpha = 0.05$ (type I error), $0.930<r<0.964$ (correlation among repeated measures), 10,000 simulations, and $n=68$ (final valid sample size). The power ($1-\beta$) exceeded 0.99; thus, the power satisfied the sufficiency requirements.

### Apparatus

The viewing distance was fixed at 57 cm from a binocular separator (SOKKIA MS16) to a colour-calibrated monitor (EIZO ColorEdge CG2730-Z; 27 inches; resolution 2560 $\times$ 1440 pixels; refresh rate 59 Hz). The binocular separator and the monitor were placed in a dark room. The experimental program was created with PsychoPy Builder (Peirce 2007; Peirce et al 2019) and controlled with a laptop (Dell G15 5511) outside the dark room. The responses were recorded using a Cedrus RB-540 response pad connected via a USB cable to a PC with the D2XX driver installed to minimize processing delays for the device using the XID 2 platform. The response keypad has only left, right, up, down, and centre buttons for use in psychological experiments that require spatial direction responses.

### Stimuli

**Fig. 1:**
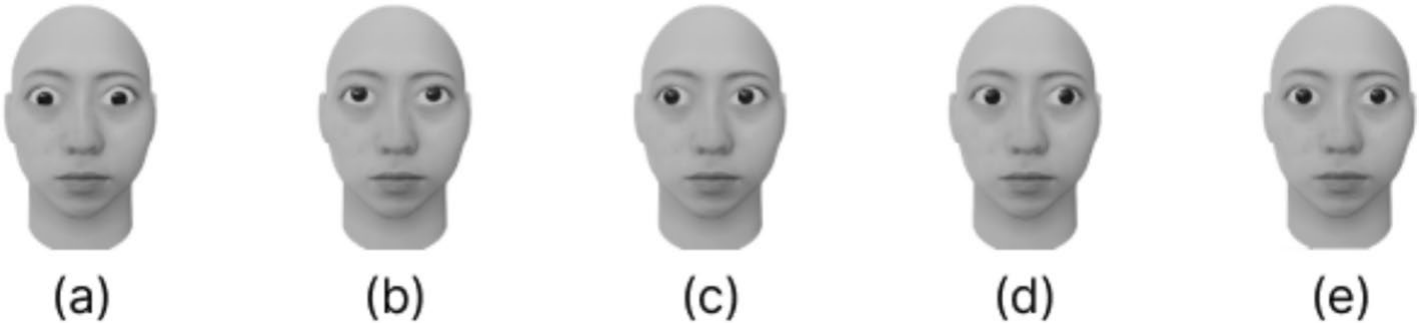
Faces with five gaze directions: (**a**) downwards, (**b**) upwards, (**c**) leftwards, (**d**) rightwards, and (**e**) direct

We created faces with five gaze directions (Fig. 1) and a dynamic Mondrian pattern.

Greyscale 3D computer-generated (CG) faces were created on the basis of a photograph of a Japanese (Asian) female using FaceGen Modeler. The 3DCG faces were chosen to create stimuli with accurate eye positions. The eyes were removed from the 3DCG faces, and 3DCG eyes (archii, Eye v3) were inserted. The gaze directions of the 3DCG faces were adjusted using Free3D and Blender (version 3.3.1) to an eccentricity of 8 degrees from the direct gaze direction to the other four directions (downwards, upwards, leftwards, and rightwards). We selected an angle of 8 degrees to introduce a subtle deviation that may be perceived as a direct gaze, particularly for a downwards gaze, on the basis of previous findings that the perceptual range of a direct gaze tends to extend more in the downwards direction (Palmer et al 2022). The size of the face was 2.5 degrees $\times$ 2.0 degrees.

A dynamic Mondrian pattern was created via the HyperText Markup Language and JavaScript. Two thousand rectangles of randomly different colours, sizes, and positions were overlaid at 100 ms intervals. Thus, the pattern was dynamically changed at 10 Hz. The size of the pattern was 5.0 degrees $\times$ 5.0 degrees.

### Procedure

The participants performed the experiment after the Miles test was completed. An experimental program was run on the basis of the participant’s dominant eye, as determined by the Miles test.

**Fig. 2:**
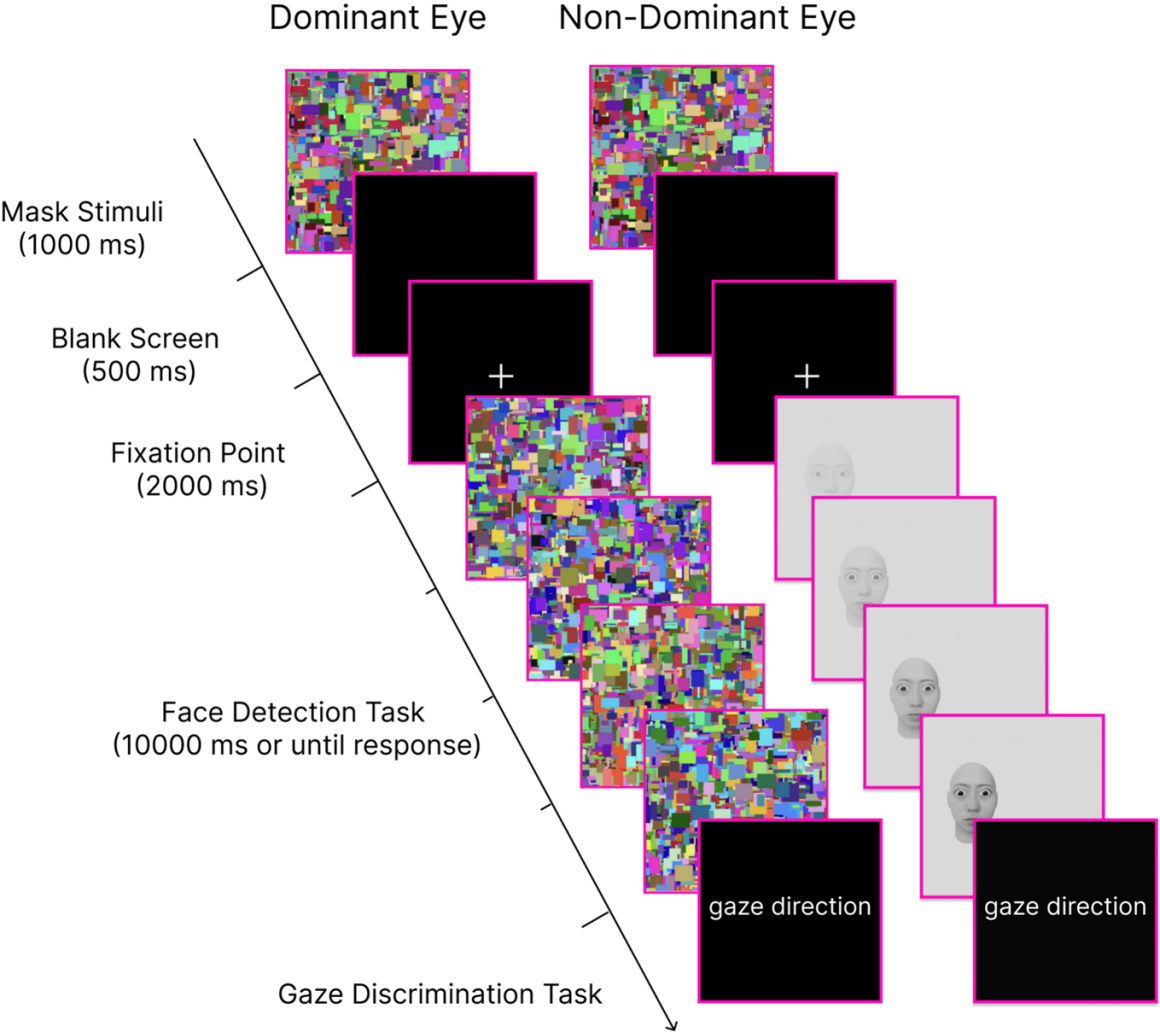
Schematic diagram of the trial in the experiment. Dynamic Mondrian patterns were presented to the dominant eye, while face stimuli were presented to the non-dominant eye. Participants observed the merged images through a binocular separator and performed two tasks: identifying whether the facial image appeared in the left or right area of the magenta square (face detection task) and categorizing the gaze direction as leftwards, rightwards, upwards, downwards, or direct (gaze discrimination task)

A schematic diagram of the trial flow is shown in the Fig. 2. First, dynamic Mondrian patterns were presented as mask stimuli in a magenta square (5.0 degree $\times$ 5.0 degree) for 1000 ms to both eyes to prevent adaptation to the visual stimuli. Second, after a blank screen was presented for 500 ms, the fixation points were presented for 2000 ms. Third, a dynamic Mondrian pattern was presented to the dominant eye, whereas the face stimulus was presented to the non-dominant eye. The face stimulus was placed inside the magenta square on either the left or right side. The transparency of the face was decreased at a constant rate over 10,000 ms from 100% (not shown) to 0% (fully shown).

The experiment consisted of two tasks (face detection and gaze discrimination) that were performed sequentially within a single-trial structure, on the basis of the procedure used by Yokoyama et al (2013). First, the participants performed a face detection task, in which they were instructed to press a key as soon as they detected a face on the left or right side of the magenta square. This task lasted for up to 10,000 ms or until a response was made. Immediately after the detection response, a gaze discrimination task began. If a face was not detected within the 10,000 ms presentation of the face and dynamic Mondrian stimuli, the participants pressed any key to proceed to the gaze discrimination task. In the gaze discrimination task, participants were asked to respond to the perceived gaze direction (downwards, upwards, leftwards, rightwards, or direct) by pressing the corresponding button. Thus, both tasks used the same face stimulus within each trial, and this trial structure was repeated throughout the experiment.

The experiment included 9 practice trials and 120 main trials. The main trials were divided into three blocks (40 trials $\times$ 3 blocks). In each block, five gaze directions were presented four times on the right and left sides of the square (5 gaze directions $\times$ 2 locations $\times$ 4 repetitions $=40$ trials). The trial sequence was pseudorandomized to ensure equal representation of all gaze directions and position combinations within each block. Breaks for a maximum of five minutes were provided between blocks.

### Analyses

The criteria for data exclusion were established as follows. Response time greater than 10,000 ms was excluded because stimulus detection in the b-CFS paradigm typically occurs within several seconds. The face stimulus was not presented beyond 10,000 ms, and any responses exceeding this duration would not reflect valid perceptual access. This cut-off is consistent with previous b-CFS studies (e.g., Lanfranco et al 2022) and was applied to ensure that only meaningful detection responses were analyzed. The mean response time for trials with the correct face position responses was subsequently calculated after excluding the data for each participant whose response time was outside the range of $\pm 2$ standard deviations from the mean in each gaze direction.

A one-way repeated-measures ANOVA was conducted on response time in the face detection task, with gaze direction as a within-subjects factor (five levels: downwards, upwards, leftwards, rightwards, and direct). Another ANOVA was also conducted on accuracy in the gaze discrimination task, with the same factor. Planned pairwise comparisons following ANOVA were performed using the Bonferroni method. Before conducting the ANOVA for response time, the normality of the data was examined using the Shapiro-Wilk test, and homoscedasticity was examined using Bartlett’s test. The confusion matrix for the gaze discrimination task was calculated to analyse the error trends for the gaze direction responses. In the planned pairwise comparisons, the *p* values were Bonferroni-adjusted by multiplying by 10, reflecting the 10 possible pairs (${}_5\textrm{C}_2$). Adjusted *p* values greater than 1 were reported as 1.000. Thus, $\alpha = 0.05$ can be directly used as a reference standard for statistical significance in all analyses.

All of the analyses were conducted using RStudio (version 2023.12.1.402, RStudio Team 2023) with R (version 4.3.3, R Core Team 2024). The Bayes factor (BF) was calculated using the BayesFactor package (version 0.9.12.4.7, Morey and Rouder 2024). The prior distribution for the ANOVA used the default in the package, a Cauchy distribution with $\gamma =0.5$ for fixed effects and $\gamma =1.0$ for random effects. The prior distribution for the *t* test was also set as per the default package, a Cauchy distribution with a scale parameter $\gamma =1/\sqrt{2}$. The strength of the evidence in the hypothesis test was evaluated using Kass and Raftery’s (1995) criteria for the value of the Bayes factor. Specifically, a ${\textrm{BF}}_{10}$ (${\textrm{BF}}_{01}$) from 1–3 indicates evidence that is *not worth more than a bare mention*, 3–20 indicates *positive* evidence, 20–150 indicates *strong* evidence, and values >150 indicate *very strong* evidence for the alternative hypothesis (the null hypothesis).

## Results

### Face detection task

To investigate gaze perception under unconscious conditions, we first aimed to determine the detection of faces displaying downwards, upwards, leftwards, rightwards, or direct gazes by measuring response time in the face detection task. The accuracy of the detected face position in the face detection task was 95.2% according to the overall data from the 68 participants. Using these filtered data, the response rate for the responses within 10,000 ms was calculated, and 91.1% of the responses were found to be valid. Subsequently, 4.0% of the overall data were excluded as outliers when the 2SD method was used. Thus, the mean response time for each participant under each condition was calculated using these valid data (87.1% of the overall data). 

We calculated the response time for each gaze direction in the face detection task. A Shapiro-Wilk test indicated that the response time data for each of the five gaze directions followed a normal distribution: downwards, $W =.976$, $p =.210$; upwards, $W =.977$, $p =.244$; leftwards, $W =.974$, $p =.170$; rightwards, $W = 0.973$, $p =.143$; and direct, $W =.967$, $p =.066$. Furthermore, the results of the Bartlett test confirmed the homoscedasticity of the data: $\chi ^2(4)=1.45$, $p=.836$. Thus, the normality and homoscedasticity of the analysis data were confirmed. 

**Fig. 3:**
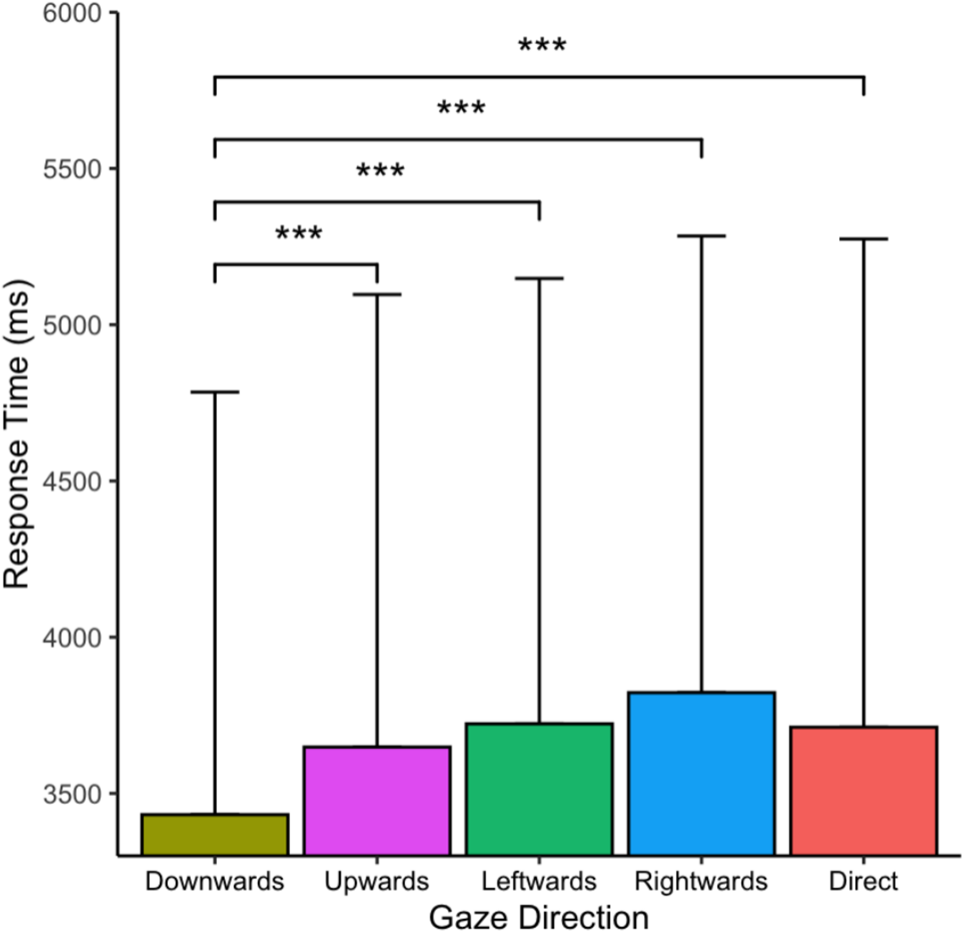
Response time in the face detection task. The error bars indicate the SDs. The asterisks indicate significant differences in the ANOVA results (*** $p_{adj} <.001$, ** $p_{adj} <.01$, * $p_{adj} <.05$)

The response time results for each gaze direction are shown in Fig. 3. The response time was $$M = 3432$$ ms ($$SD = 1352$$) in the downwards, $$M = 3648$$ ms ($$SD = 1448$$) in the upwards, $$M = 3723$$ ms ($$SD = 1425$$) in the leftwards, $$M = 3822$$ ms ($$SD = 1461$$) in the rightwards, and $$M = 3712$$ ms ($$SD = 1562$$) in the direct gaze directions. The ANOVA results revealed that the main effect of the gaze direction on the response time was statistically significant, $$F(4, 268)= 12.63, MSE = 10.08, p<.001$$ ($$\eta_p^2 =$$$$ 0.159, \rm{BF}_{10}=50.06\times10^{5}$$). The BF value indicated *very strong* evidence for the alternative hypothesis. Planned pairwise comparisons revealed significant differences with *very strong* evidence for the alternative hypothesis between the downwards gaze direction and upwards gaze direction, $$t(67) = -4.15, $$$$ p_{adj} <.001$$ ($$d_{z} = -0.50,$$$$ \rm{BF}_{10}=217$$), the downwards and leftwards gaze directions, $$t(67)$$$$ = -5.78, p_{adj} < .001$$ ($$d_{z} $$$$ = -0.70, \rm{BF}_{10}=65.22\times10^{3}$$), the downwards and rightwards gaze directions, $$t(67) = -6.62, p_{adj} < .001$$ ($$d_{z} = -0.80,$$$$ \rm{BF}_{10}$$$$ =16.27\times10^{5}$$), and the downwards and direct gaze directions, $$t(67) = -4.57, $$$$ p_{adj} < .001$$ ($$d_{z} = -0.55, $$$$\rm{BF}_{10}$$$$=861$$). The other six pairs were not significantly different: upwards and leftwards: $$t(67) = -1.58, p_{adj} = 1.000$$ ($$d_{z} =$$$$ -0.19, \rm{BF}_{01}=2.29$$); upwards and rightwards: $$t(67) = -2.74, p_{adj} = .078$$ ($$d_{z} = -0.33,\rm{BF}_{10}=4.15$$); upwards and direct: $$t(67) = -1.01, p_{adj} = 1.000$$ ($$d_{z} = -0.12, $$$$\rm{BF}_{01}=4.61$$); leftwards and rightwards: $$t(67) = -1.79, p_{adj} = .781$$ ($$d_{z} = -0.22, \rm{BF}_{01}=1.67$$); leftwards and direct: $$t(67) = 0.20, p_{adj} = 1.000$$ ($$d_{z} = 0.02, \rm{BF}_{01}=7.37$$); and rightwards and direct: $$t(67) = 1.59, p_{adj} = 1.000$$ ($$d_{z} = 0.19, \rm{BF}_{01}=2.29$$). The results revealed that the pairs of “upwards and direct” and “leftwards and direct” pairs provided *positive* evidence in support of the null hypothesis; the “upwards and rightwards” pairs indicated *positive* evidence in support of the alternative hypothesis; and the other three pairs provided the evidence of *not being worth more than a bare mention* for either the null or alternative hypotheses. In summary, compared with the response time for the other four gaze directions using planned pairwise comparisons, the response time for the downwards direction was shorter. These results suggest that, compared with other gaze directions, a downwards gaze plays an important role in unconscious and conscious processing compared to other gaze directions.

### Gaze discrimination task

**Fig. 4:**
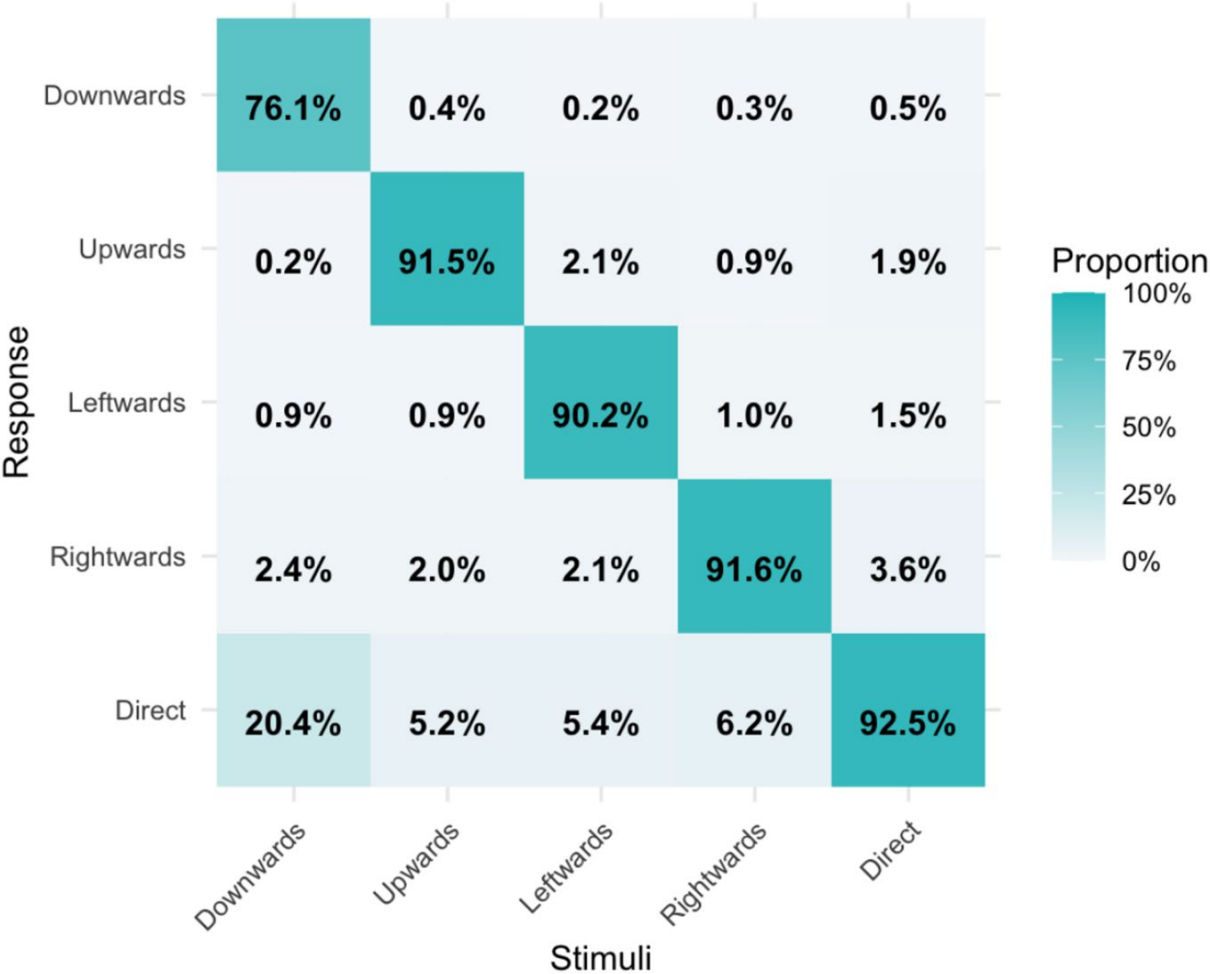
Confusion matrix graph of the gaze discrimination task. The *y*-axis represents the predicted categories of the gaze direction (response), whereas the *x*-axis represents those of the actual categories (stimuli). The diagonal component indicates the rate of correct responses. The nondiagonal component indicates the rate of incorrect responses

To investigate how face stimuli were perceived, the responses in the gaze discrimination task were compared with the stimuli that were actually presented. The ANOVA results comparing the correct response rate for each face stimulus revealed significant differences among the different gaze directions, $$F(4, 268)= 12.65, MSE = 0.08, p<.001$$ ($$\eta_p^2 = 0.159,$$$$ \rm{BF}_{10}=62.50\times10^{5}$$). The BF value indicated *very strong* evidence in support of the alternative hypothesis. The planned pairwise comparison revealed that the correct response rate for the downwards gaze was lower than was that for the upwards gaze, $$t(67) = -4.13, p_{adj} = .001$$ ($$d_{z} = -0.50,$$$$ \rm{BF}_{10}=205$$), the leftwards gaze, $$t(67) = -3.87, $$$$p_{adj} = .003$$ ($$d_{z} = -0.47, $$$$ \rm{BF}_{10}=89.71$$), the rightwards gaze, $$t(67) = -4.46, $$$$ p_{adj} < .001$$ ($$d_{z} = -0.54, \rm{BF}_{10}=609$$), and the direct gaze, $$t(67) = -4.47, p_{adj} < .001$$ ($$d_{z} = -0.54,$$$$ \rm{BF}_{10}=625$$). All of these comparisons provided *very strong* evidence for the alternative hypothesis. However, the other six pairs were not significantly different with *positive* evidence in support of the null hypothesis: upwards and leftwards: $$t(67) = 0.74, p_{adj} = 1.000$$ ($$d_{z} = 0.09, $$$$ \rm{BF}_{01}$$$$ =5.76$$); upwards and rightwards: $$t(67) = -0.06,$$$$ p_{adj} = 1.000$$ ($$d_{z} = -0.01,$$$$ \rm{BF}_{01}$$$$ =7.50$$); upwards and direct: $$t(67) = -0.61, p_{adj} = 1.000$$ ($$d_{z} = -0.07, $$$$\rm{BF}_{01}$$$$=6.29$$); leftwards and rightwards: $$t(67) = -0.69, p_{adj} = 1.000$$ ($$d_{z} = -0.08, \rm{BF}_{01}=5.99$$); leftwards and direct: $$t(67) = -1.26, p_{adj} = 1.000$$ ($$d_{z} = -0.15,$$$$\rm{BF}_{01}$$$$=3.55$$); and rightwards and direct: $$t(67) = -0.40, p_{adj} = 1.000$$ ($$d_{z} = -0.05, \rm{BF}_{01}=6.97$$). When the correct response rates for the categories of the five gaze directions were compared, the correct response rate for the downwards direction was lower than was that for the other directions (Fig. 4). Approximately 20.4% of the downwards gazes were reported to be the direct gazes. These results indicate that a downwards gaze can sometimes be misinterpreted as a direct gaze.

## Discussion

The aim of this study was to investigate the effect of the gaze direction on face awareness under the b-CFS paradigm. The results revealed that faces with a downwards gaze were perceived faster than faces with the other four gaze directions were (upwards, leftwards, rightwards, and direct). Additionally, the rate of correct responses for the 8-degree downwards gaze direction was lower than was that for the other gaze directions. These findings suggest that faces with a downwards gaze are more likely to be processed subconsciously and that the gaze direction is less likely to be accurately perceived. The present findings suggest that downwards gazes function as a social stimulus, distinct from other averted gaze directions.

The results of the present study revealed for the first time that downwards gazes are more likely to be detected than are averted gazes with other gaze directions when a b-CFS paradigm is used. Previous studies have revealed that, under a b-CFS paradigm, faces with direct gazes were detected more quickly than were those with leftwards or rightwards gazes (Chen and Yeh 2012; Seymour et al 2016; Stein et al 2011). However, while these studies concluded that direct gazes are more readily perceived than averted gazes are, upwards and downwards gaze directions were not examined in previous studies. The enhanced detection of a downwards gaze found in the present study suggests a unique mechanism underlying its processing. One possible explanation for this finding is the role of self-referential processing (Baltazar et al 2014; Conty et al 2016; Hietanen 2018). The direction of a downwards gaze is oriented towards the face and body, potentially resulting in higher self-referential processing than the four other gaze types. However, the interpretation involving self-referential mechanisms remains speculative in this study. To provide stronger support for the self-referential hypothesis, future research should combine the current paradigm with a psychophysical estimation of the cone of direct gaze (e.g., Gamer and Hecht 2007), thereby allowing a direct examination of whether a downwards gaze is interpreted as self-directed. In addition, combining the current paradigm with a self-relevance task (e.g., Macrae et al 2017) would allow researchers to investigate directly whether individuals with heightened self-referential processing detect downwards gazes more rapidly. Taken together, these approaches would provide further support for the involvement of self-referential mechanisms in the awareness of faces with downwards gazes.

The present study did not reveal any significant differences in the accessibility or detectability of faces with upwards, rightwards, leftwards, or direct gazes in the face detection task. These findings suggest the failure to replicate the previous findings that direct gazes are perceived earlier than rightwards and leftwards gazes are under a b-CFS paradigm (Chen and Yeh 2012; Seymour et al 2016; Stein et al 2011). One possible explanation for this discrepancy lies in the distinct deviation angle of the gazes among the present and previous studies. Specifically, we employed a relatively small gaze deviation of 8 degrees, which is included in the range perceived as a direct gaze depending on the measurement method (e.g., Balsdon and Clifford 2018; Cline 1967). In psychophysical studies that manipulate gaze direction, the deviation angle of the gaze direction is typically reported in detail (e.g., Gamer and Hecht 2007; Mareschal et al 2013). In contrast, several b-CFS studies describe gaze direction only qualitatively, using terms such as “direct” or “averted” without specifying the exact angle. Nevertheless, on the basis of the visual appearance of the stimuli, the averted gazes in those studies appear to involve larger deviations than those used in the present study. Indeed, some previous studies using (b-)CFS paradigms have reported and used considerably larger gaze deviations for averted gazes (e.g., 20 degrees in Jackson et al, 2022; 31.6 degrees in Stein et al, 2012). Thus, the absence of a significant difference between direct and averted gazes in this study may be attributed to the relatively small deviation angle used. These findings highlight the importance of precisely defining gaze direction when the unconscious processing of gaze under interocular suppression is investigated.

The results of the gaze discrimination task in the present study revealed a perceptual ambiguity: a downwards gaze was frequently interpreted as a direct gaze. Perceptual ambiguity was also demonstrated by Palmer et al (2022), who reported that the focal point of eye contact tends to shift downwards. These findings highlight the complexity of vertical gaze processing and its influence on social perception. Its enhanced detectability, combined with its perceptual ambiguity, underscores the importance of considering vertical gaze variations in studies of gaze perception.

This study has three main limitations. First, the participants performed the face detection and gaze discrimination tasks in one trial. In general, performance differs depending on which task is prioritized under dual-task conditions (Pashler 1994; Seli et al 2013; Standage et al 2014). The finding that faces with downwards gazes were more easily identified and misperceived under the b-CFS paradigm may be considered a trade-off in this dual task paradigm for responses to faces with downwards gazes. Indeed, a previous study suggested that the detection process may precede higher level classification processes in CFS (Kobylka et al 2017). Furthermore, Pournaghdali and Schwartz (2020) reported that the methods used to measure consciousness differ across various studies, and it is unclear how procedural differences affected the present results. Given these limitations, future studies should conduct additional experiments to separate the contributions of perceptual detection and gaze discrimination by dividing the two tasks into independent blocks or reversing the task order. Second, this study used only a single 3DCG face derived from a photograph of an Asian female. The specific nature of the stimuli may compromise the ecological validity and limit the generalizability of the findings to a narrower range of facial identities. Indeed, previous studies have reported that the effects of gender and race on facial features affect face awareness under the b-CFS paradigm (Amihai et al 2011; Caruana et al 2019; Stein et al 2014). Future studies should include multiple facial identities that vary in gender and race to assess the robustness of the effects of a downwards gaze across a broader range of social and perceptual variables. Third, a slight downwards gaze may be perceived as emotionally salient despite the fact that our face stimuli are neutral in expression. Previous studies have shown that gaze direction interacts with facial expressions in face processing and social cognition as demonstrated in various cognitive tasks (e.g., Adams Jr and Kleck 2005; Graham et al 2010; Rigato and Farroni 2013). In particular, a downwards gaze has been suggested to be perceived as a facial expression of contempt (Gervais and Fessler 2017) or sadness (Semyonov et al 2021). In a b-CFS paradigm, facial expressions have been shown to modulate suppression breakthrough (Stewart et al 2012; Yang et al 2007) and to affect face awareness, surpassing even the effects of gaze (Caruana et al 2019). Therefore, future studies should explicitly examine the emotional interpretations of gaze direction to clarify the role of a downwards gaze in modulating visual awareness.

In summary, the results of this study suggest that a slight downwards gaze may draw preferential attention during unconscious processing and may occasionally be misperceived as direct gaze, although this interpretation should be regarded as tentative. This result may be due to the watching eye effect (e.g., Conty et al 2016), the perception of being gazed at (e.g., Palmer et al 2022), or emotional meanings (e.g., Semyonov et al 2021). However, since gaze is used in many forms of social communication and has various meanings, these results are difficult to generalize. On the basis of these results, future research should further elucidate the mechanisms through which slightly averted or downwards gazes function as a facet of social cognition.
